# Variation in dengue virus plaque reduction neutralization testing: systematic review and pooled analysis

**DOI:** 10.1186/1471-2334-12-233

**Published:** 2012-09-28

**Authors:** Kaitlin Rainwater-Lovett, Isabel Rodriguez-Barraquer, Derek AT Cummings, Justin Lessler

**Affiliations:** 1 Department of Epidemiology, Johns Hopkins Bloomberg School of Public Health, Baltimore, Maryland, USA

## Abstract

**Background:**

The plaque reduction neutralization test (PRNT) remains the gold standard for the detection of serologic immune responses to dengue virus (DENV). While the basic concept of the PRNT remains constant, this test has evolved in multiple laboratories, introducing variation in materials and methods. Despite the importance of laboratory-to-laboratory comparability in DENV vaccine development, the effects of differing PRNT techniques on assay results, particularly the use of different dengue strains within a serotype, have not been fully characterized.

**Methods:**

We conducted a systematic review and pooled analysis of published literature reporting individual-level PRNT titers to identify factors associated with heterogeneity in PRNT results and compared variation between strains within DENV serotypes and between articles using hierarchical models.

**Results:**

The literature search and selection criteria identified 8 vaccine trials and 25 natural exposure studies reporting 4,411 titers from 605 individuals using 4 different neutralization percentages, 3 cell lines, 12 virus concentrations and 51 strains. Of 1,057 titers from primary DENV exposure, titers to the exposure serotype were consistently higher than titers to non-exposure serotypes. In contrast, titers from secondary DENV exposures (n = 628) demonstrated high titers to exposure and non-exposure serotypes. Additionally, PRNT titers from different strains within a serotype varied substantially. A pooled analysis of 1,689 titers demonstrated strain choice accounted for 8.04% (90% credible interval [CrI]: 3.05%, 15.7%) of between-titer variation after adjusting for secondary exposure, time since DENV exposure, vaccination and neutralization percentage. Differences between articles (a proxy for inter-laboratory differences) accounted for 50.7% (90% CrI: 30.8%, 71.6%) of between-titer variance.

**Conclusions:**

As promising vaccine candidates arise, the lack of standardized assays among diagnostic and research laboratories make unbiased inferences about vaccine-induced protection difficult. Clearly defined, widely accessible reference reagents, proficiency testing or algorithms to adjust for protocol differences would be a useful first step in improving dengue PRNT comparability and quality assurance.

## Background

The re-emergence and geographic expansion of dengue virus (DENV) over the past several decades has resulted in the infection of 50–500 million individuals each year [1,2]. Several rapid diagnostic tests and enzyme-linked immunosorbent assays have been developed for the detection of serologic immune responses to DENV exposure [3], but the plaque reduction neutralization test (PRNT) remains the gold standard. The PRNT requires antibodies to neutralize and prevent virions from infecting cultured cells, and is believed to represent a protective antibody response. While the basic concept of the PRNT remains constant, this test has evolved in multiple laboratories throughout the world since its development [4], introducing variation in methods that may influence the comparability of results. For example, cell type, virus passage, and the use of complement were previously identified as sources of variation and had varying effects between serotypes [5]. Additionally, higher plaque neutralization levels (e.g., 90% vs. 50%) have demonstrated less sensitivity [6].

Researchers and the World Health Organization’s (WHO) Task Force on Clinical Trials of Dengue Vaccines have proposed standardizing the dengue PRNT through the use of reference sera or proficiency panels [7-9]. Recently, Roehrig et al. (2008) and the WHO (2009) recommended specific dengue PRNT guidelines to encourage standardization across laboratories for comparison of DENV vaccine trials [7,10,11]. As efforts to develop DENV vaccines expand, more detailed characterizations of antibody responses and factors influencing the measurement of these responses are essential. Although several vaccine candidates are under development, comparisons between laboratories will be difficult without relatively standardized laboratory materials and methods [12] or algorithms to control for variations in protocol. Examples of this variation include the addition of complement or reporting sera dilutions before or after the addition of virus. How different strains relate to one another with respect to antibody cross-neutralization has implications for the type of strains to be included in vaccine suspensions, whether a global or a region-specific vaccine should be used, as well as future research efforts after the development of a successful vaccine.

The objectives of this study were to characterize the extent of variation in laboratory materials and methods and the effects of this variation on PRNT titers using published data. Here, we present a systematic review of literature reporting individual-level PRNT titers to identify factors associated with heterogeneity in PRNT results and compare variation between strains within DENV serotypes and between articles (to capture potential inter-laboratory differences) using hierarchical models.

## Methods

### Search strategy and selection criteria

A systematic review was conducted during May-June 2009 using the PubMed and ISI Web of Knowledge 4.0 databases. Nine searches were performed using the search term “dengue” and each of the following terms or phrases: “antibody”, “serology”, “neutralization”, “serum neutralization”, “prnt”, “infection history”, “previous infection”, “longitudinal”, “antigenicity”, “vaccine”, “chimerivax” and “over time”. Articles were not restricted by year of publication and included electronic, ahead-of-print publications available in these databases. We did not attempt to contact authors to obtain unpublished PRNT titers. Abstracts were screened by two individuals (KRL and IRB). Articles in languages other than English, Spanish, and Portuguese and case reports or studies of unusual patient populations were excluded. Unusual patient populations were defined as individuals with co-morbidities (e.g., cancer or organ failure) or neurologic or central nervous system manifestations. Articles that did not report dengue PRNT titers from humans or did not state the time from dengue virus exposure to sera collection were also excluded.

### Assessment

Each article was categorized based on the type of sera samples collected. Natural exposure studies included serological surveys (sera collected from individuals regardless of dengue-like symptoms), infection studies (sera collected in response to suspected dengue infection as determined by study investigators or clinic/hospital staff), and human inoculation studies or accidental inoculations in a laboratory setting. Vaccine studies were defined as the collection of sera samples after enrollment in a DENV vaccine study.

### Data abstraction

Titers against each of the four DENV serotypes were recorded from individual human subjects and classified as natural exposure or vaccination. Titers reported in tables or the text were abstracted while titers illustrated in figures or requiring calculations by abstractors (e.g., an index score) were excluded. For the descriptive analysis, minimum titers (e.g., < 1:10 or < 1:40) were set to zero and end-point titers that exceeded the maximum dilution specified in the article, such as ≥ 1:2560 or ≥ 1:5120, were set to that value. The recorded materials and methods used in PRNTs for each article included dengue serotypes and strains, virus concentrations, cell lines, and neutralization percentages (required percentage of plaques neutralized). If possible, authors were contacted via email to obtain information on the DENV strains used for PRNT; however, other raw data were not requested.

The approximate time from infection or vaccination to serum sample collection was categorized as unexposed, acute (< 12 days), convalescent (12–30 days), late convalescent (30 days–1 year), and very late convalescent (> 1 year) [13,14]. If a range of time was reported for an individual sera sample (e.g., “Serum was collected 6–12 months following infection…”), the mid-point between the minimum and maximum of the range was used. If acute and convalescent sera samples were collected but exact times from exposure or symptom onset were not reported, acute samples were defined as 4 days post-exposure and convalescent samples defined as 11 days post-exposure. If time from symptom onset to serum sample collection was reported, 5 days were added to account for the 4–6 day incubation period of dengue virus [13] and make times since exposure comparable between post-symptom onset and post-exposure reports.

Previous DENV exposure or infection (primary/secondary/tertiary) was based on explicit report by the authors. “Confirmed” exposure to a specific serotype was based on the reported contents of a vaccine in a vaccine study or inoculation suspension in inoculation studies or a polymerase chain reaction (PCR)-confirmed infection in natural exposure studies. If these criteria were not met, the probable infecting serotype was noted by abstractors but not considered confirmed. For example, probable infecting serotypes were commonly deduced by an article’s authors from changes in serotype-specific antibody titers or the most frequently detected serotype during a recent epidemic.

### Pooled analysis

Pooled analyses were conducted using PRNT titers for which a confirmed exposure serotype and exposure history (e.g., uninfected, primary, or secondary) could be determined. These analyses aimed to characterize the effects of strain choice and inter-laboratory variation on PRNT titers after adjusting for laboratory methods, exposure route and time since exposure. In the case of primary exposure, it was assumed that natural logarithm-transformed (log) titers depended on the exposure serotype. For secondary exposure it was assumed that log titers were independent of the exposure serotype, as indicated by the descriptive analysis of this data and prior studies [15]. Pooled median log titers for each exposure-testing serotype were calculated as a weighted mean of strain-specific medians with the number of strain-specific observations as weights.

We constructed log-linear hierarchical models to characterize the effects of strain choice and inter-laboratory variation on titers. Titers from individuals who were simultaneously exposed to two or more serotypes via multitypic vaccination were excluded. Titers reported as less than the minimum dilution for that particular article (e.g., <1:10 or <1:40) were treated as censored observations distributed between 0.1 and the minimum reported titer in that article. The maximum reported titers were also treated as censored observations distributed between the maximum reported titer and a 100-fold increase.

Model A assessed the effect of strain choice on log PRNT titer. Categorical strain variables were assumed to have random effects with mean zero and estimated variance. Strains originating in Thailand were used as the reference strain within each serotype due to frequent use in the abstracted articles and to provide geographic consistency. Strains reported by only one article were categorized as “Other” in each serotype. The strain categories were as follows: DENV1 – 16007, Hawaii, Other; DENV2 – 16681, New Guinea B, New Guinea C, PR-159, Other; DENV3 – 16562, H87, 116/00, Other; DENV4 – 1036, H241, Dominica /814669, 4328S, Other. Model B assessed the effect of inter-laboratory variation on log PRNT titer. A random effect for article, with zero mean and estimated variance, was included in this model. Due to collinearities between the strain choice and article, a model with effects for both strain and article was not evaluated.

In both models fixed effects were estimated to account for infecting serotype and other factors that might affect measured PRNT titer. For primary exposures, intercepts were estimated for each exposure serotype-testing serotype pair while for secondary exposures, intercepts were estimated for each testing serotype. The following were also evaluated during model selection: time since exposure, neutralization percentage, cell line, virus concentration and vaccination. Neutralization percentage estimates the effects of each 10% increase in neutralization percentage after adjusting for other covariates. Similarly, the effect of vaccination is estimated with reference to individuals with natural exposure after adjusting for other covariates. Time since exposure was treated as an ordinal variable using categories described above with unexposed sera as the reference category.

All models were fit using Monte Carlo Markov Chain (MCMC) methods in OpenBUGS [16] and the R Statistical Package (version 2.10, R Development Core Team, Vienna, Austria). Standard non-informative priors were used for all fixed effects and estimated variance components. For each model, three chains of 10,000 iterations were run. The final 5,000 iterations of all chains were combined to estimate the posterior distribution of parameters. Models were selected based on minimizing the deviance information criteria (DIC). The mean of the posterior distribution is reported as the parameter estimate, and 90% credible intervals report the range between the 5^th^ and 95^th^ percentiles of the posterior distribution. Convergence was assessed by examination of the MCMC chains and R-hat values for all estimated parameters of <1.1 [17]. Full specifications of the models are in Additional File 1.

## Results

**Figure 1:**
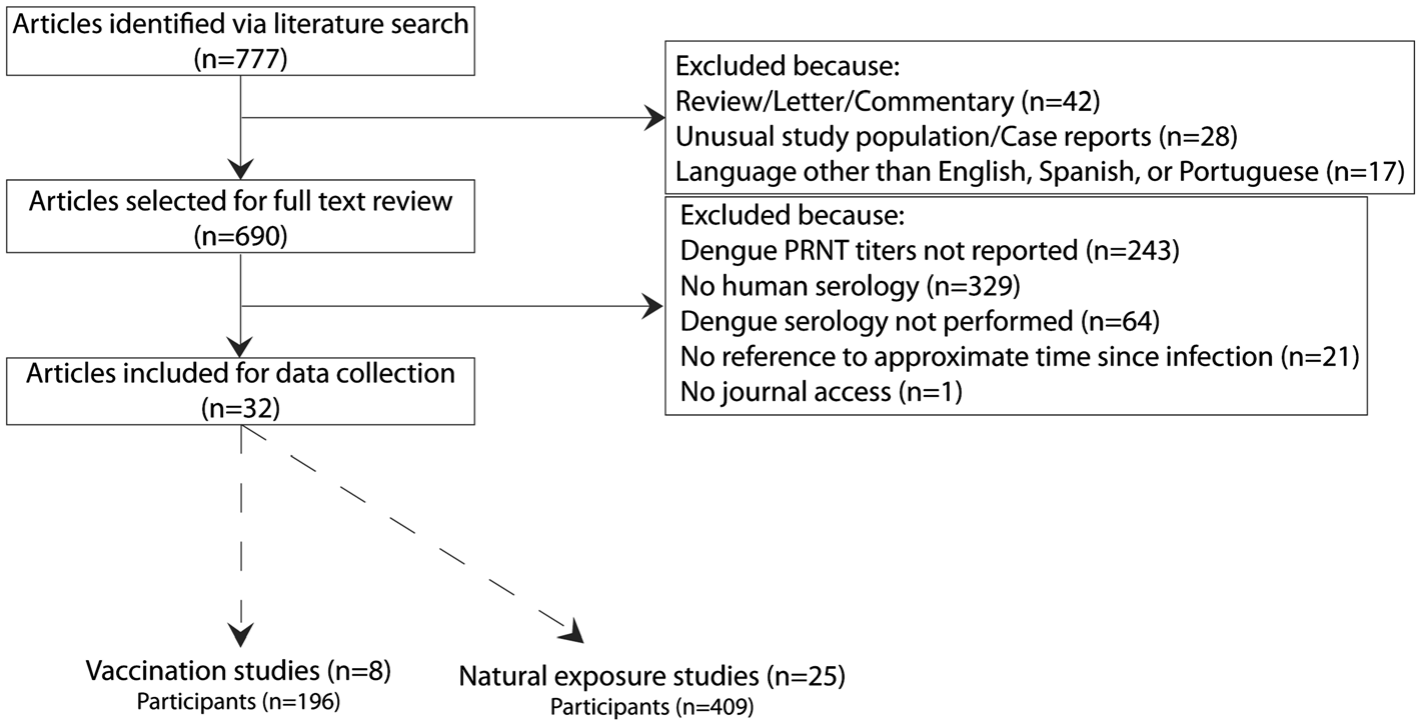
**Article selection and inclusion/exclusioncriteria.** Articles do not sum to 32 as one report included multiple exposure routes and is included in both exposure categories

The literature searches yielded 777 articles. Eighty-seven articles were eliminated after abstract screening and 658 additional articles were eliminated after full text review (Figure 1). Of the 17 articles excluded due to non-English/Spanish/Portuguese language, the languages of publication were Chinese (n = 2), Danish (n = 1), French (n = 6), German (n = 5), and Japanese (n = 3). Sources of variation among the 32 articles meeting the inclusion criteria included geographic variation of the study population, neutralization percentage, cell line, virus concentration, and strain (Tables 1 and 2). The 32 articles reported 4,411 PRNT titers from 605 human subjects (Table 3). Twenty-five articles (78%) reported data from natural exposure studies and eight (25%) described vaccine studies. Articles do not sum to 32 because one article reported titers resulting from vaccination and natural exposure (Table 1) [18].

**Table 1:** **Number of articles reportingsubject and laboratory characteristicsstratified by exposure route**

	**Vaccination**	**Natural Exposure**	**Total**
*Geographic location of studypopulation*			
Asia	2	15	17
North America	5	11	15*
South America	0	1	1
Africa	0	0	0
Australia	1	0	1
Europe	0	2	2
Not reported	1	1	1*
*Neutralization percentage*			
50 %	5	18	23
70 %	0	3	3
80 %	2	2	3*
90 %	0	1	1
Not reported	1	2	3
*Cell line*			
BHK	0	9	9
LLC-MK_2_	5	11	16
Vero	3	4	6*
Not reported	0	2	2
*Virus concentration (plaque-forming units[pfu] or focus-forming units[ffu] per microliter [uL]or milliliter [mL])*			
10-20 pfu / 10 uL	0	1	1
10-20 pfu / 50 uL	0	2	2
15-25 pfu / 25 uL	0	2	2
20-30 pfu / 12.5 uL	0	2	2
40-80 pfu / 100 uL	0	1	1
50 pfu / 150 uL	3	4	7
10^7^ pfu / mL	2	0	2
25-50 pfu	0	1	1
50 pfu	0	1	1
320 pfu	0	1	1
100 ffu / 90 uL	0	1	1
800 ffu	1	1	1*
Not reported	2	8	10

* indicates articles do not sum in row as Vasilakis et al. [18] reported titers from vaccination and natural exposure.

**Table 2:** **Number of articles reportingspecific strains stratified byexposure route**

	**Vaccination**	**Natural Exposure**	**Total**
*DENV1 strain*			
Hawaii	1	8	9
16007	2	4	6
Mochizuki	0	2	2
Other	2	4	5*
Not reported	2	7	9
n/a	1	2	3
*DENV2 strain*			
New Guinea B	0	3	3
New Guinea C	1	3	4*
16681	3	6	8*
PR-159	1	1	2
Other	2	4	6
Not reported	2	6	8
n/a	0	2	2
*DENV3 strain*			
H87	0	9	9
116/00	0	2	2
16562	2	4	6
CH53489	2	0	2
PR-6	0	2	2
Other	1	3	4
Not reported	2	7	9
n/a	1	0	1
*DENV4 strain*			
H241	1	9	9*
Dominica	1	1	2
4328-S	0	3	3
1036	2	0	2
Other	2	2	4
Not reported	2	6	8
n/a	1	4	5

* indicates articles do not sum in row as Vasilakis et al. [18] reported titers from vaccination and natural exposure.

**Table 3:** **Summary of articles meetingthe inclusion criteria**

**Author & Publication Year(Reference)**	**Study Population**	**Age [years]**	**# Subjects (# Titers)**	**Days Post- Exposure**	**Neut. %**	**DENV1 strains**	**DENV2 strains**	**DENV3 strains**	**DENV4 strains**
***Natural Exposure Studies***									
Russell et al. 1967 [19]	Thailand, USA	children, adults	7 (28)	17-28	50	--	--	--	--
Sukhavachana et al. 1969 [20]	--	--	2 (16)	7, 14	50	Hawaii	New Guinea C	H87	H241
Scott et al. 1972 [21]	Puerto Rico	--	3 (11)	11-20	50	Hawaii	PR-109	H87	23751
Russell and McCown 1972 [22]	Puerto Rico, Tahiti	--	5 (30)	21	50	n/a	n/a	H87, 21153, PR-6, PR-38, J-1007, Tahiti-4	n/a
Halstead 1974 [23]*	USA	adults	9 (36)	15341-17532	50	16007	16681	16562	4328-S
Papaevangelou and Halstead 1977 [24]*	Greece	adults	45 (180)	16436	50	16007	16681	16562	4328S
Fujita and Yoshida 1979 [25]	Japan	45-74	9 (36)	12419	50	Mochizuki, Hawaii	Trinidad 1751	H87	H241
Scott et al. 1976 [26]	Thailand	children	6 (48)	3-67	50	--	--	--	--
van Peenen et al. 1978 [27]	Indonesia	1-18	4 (32)	7, 14	--	--	--	--	--
Okuno et al. 1980 [28]*	Thailand	1-13	20 (160)	1-13	50	Hawaii	New Guinea B	H87	H241
Fukunaga et al. 1983 [29]*	Japan	28-55	9 (36)	2-13	50	Hawaii	New Guinea B	H87	H241
Okuno et al. 1983 [30]	Japan	adults	11 (51)	1826-14610	--	Hawaii, Mochizuki	New Guinea B	H87	H241
Sangkawibha et al. 1984 [15]*	Thailand	1-8	8 (60)	0-17	50	16007	16681	16562	4328-S
Morens et al. 1985 [31]	Puerto Rico	--	24 (267)	0-70	50 & 70	CV.1636 / 77	PR-159	PR-6	n/a
Rosen 1986 [32]*	Greece, Pacific Islands, USA	42-60	68 (268)	7-16436	90	Hawaii	New Guinea C	H87	H241
Kliks et al. 1989 [33]*	Thailand	children	10 (40)	0	50	--	D80-616	--	--
Kuno et al. 1993 [34]	Puerto Rico	4-50	9 (79)	0-83	50	--	--	--	--
Graham et al. 1999 [35]*	Indonesia	4-9	17 (131)	0, 10-361	70	16007	16681	16562	1009
Yamada et al. 2003 [36]	Japan	--	37 (71)	2-44	50	--	--	--	--
Alvarez et al. 2006 [37]*	Cuba	--	43 (371)	3-21, 517-834	50	Angola	A15/81, I348600	116/00	Dominica / 814669
Alvarez et al. 2008 [38]*	Cuba	--	20 (140)	515	50	n/a	n/a	116/00, 118/00, 140/00, 167/01, 557/01, 21/02, Puerto Rico	n/a
Lai et al. 2008 [39]*	Taiwan	--	7 (32)	7-14	70	Hawaii	New Guinea C	H87	H241
da Silva-Nunes et al. 2008 [40]	Brazil	5-90	20 (120)	0, 274	50	--	--	--	n/a
Crill et al. 2009 [41]*	Taiwan, Puerto Rico	--	12 (48)	6-18	80	56BC94 / 95	16681	116RC1396	H241
***Vaccination Studies***									
Summers et al. 1984 [42]*	USA	--	8 (16)	30, 180	50	n/a	PR-159	n/a	n/a
Rabablert et al. 2000 [43]*	Thailand	18-35	8 (64)	0, 60	--	16007	16681	16562	1036
Rothman et al. 2001 [44]	USA	--	6 (48)	60, 180	80	Hawaii	New Guinea C	CH53489	Dominica / 814669
Kanesa-Thasan et al. 2003 [45]*	USA	18-49	20 (397)	0, 28, 60, 120, 180	50	--	--	--	--
Guy et al. 2004 [46]	Thailand	4-14	16 (128)	273, 2009	50	16007	16681	16562	1036
Kitchener et al. 2006 [47]	Australia	21-39	10 (40)	42	50	--	--	--	--
Vasilakis et al. 2008 [18]*^	USA, unknown location	18-50	61 (719)	42 vacc, 16 infect	80	OBS7690, P72-1244	16681, 1349, IQT1950, 1328, P8-1407, DakArA510, DakArA1247, DakArA2022	FSP-032	H241, P75-125
Sun et al. 2009 [48]	USA	18-45	71 (704)	28, 180, 208	50	45AZ5	S16803	CH53489	341750

“Neut. %” indicates neutralization percentage; “DENV#” indicates dengue virus serotype; “—” indicates unreported data; “n/a” indicates serotype was not tested, ^ indicates article contains titers from vaccination and infection studies, * indicates article was included in the pooled analysis.

**Figure 2:**
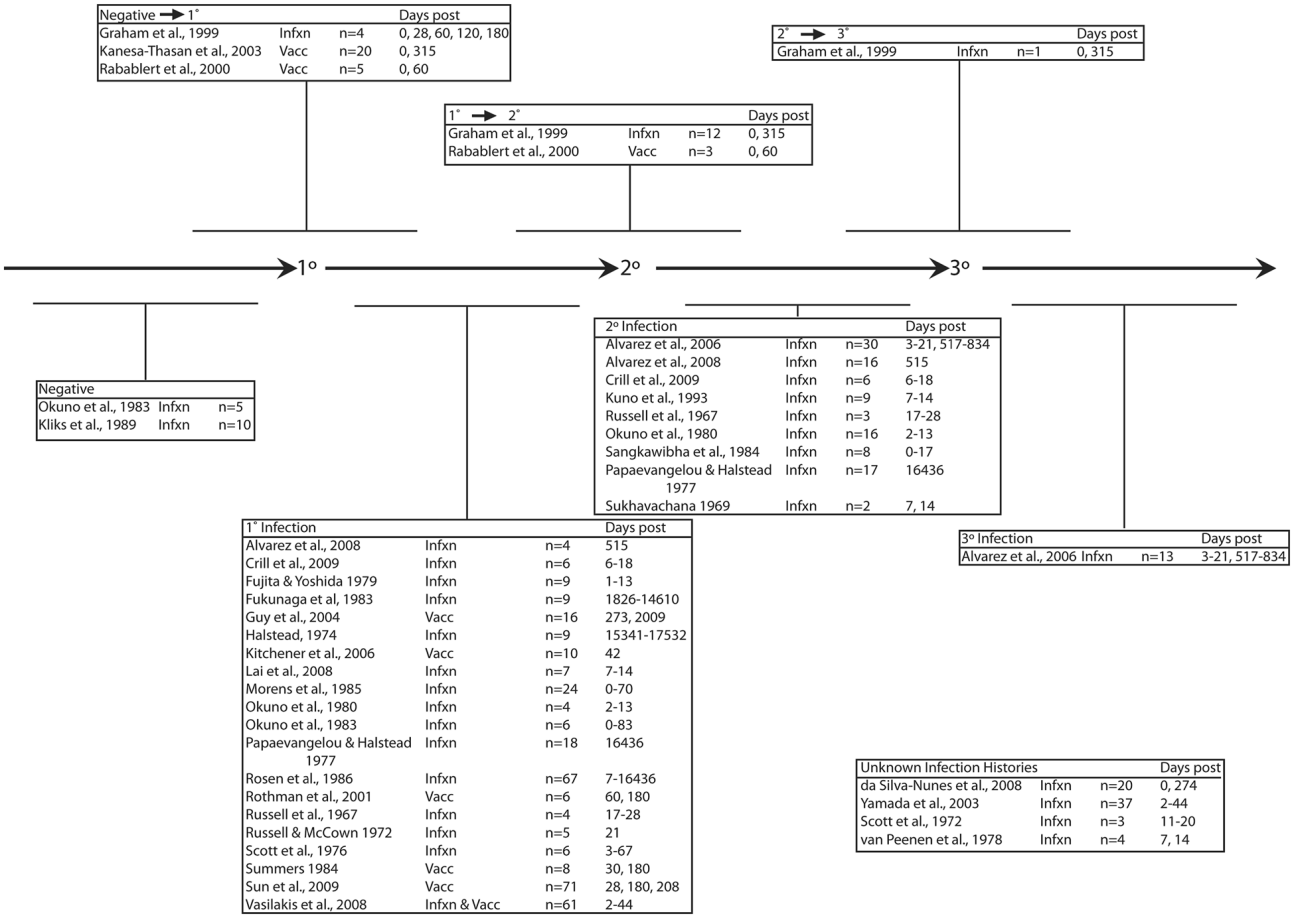
**Dengue virus (DENV) exposuretimeline of PRNT titersincluded in this review.** Articles above the timeline reported transitions between exposure states while articles below reported single states. 1°, 2°, and 3° abbreviate primary, secondary, and tertiary exposures; “n” indicates the numbers of individuals who contributed titers that met inclusion and exclusion criteria; “Days Post” indicates the number of days between DENV exposure and serum collection.

The majority of studies reported titers at one or two time points following primary or secondary DENV exposure and few reports described changes in titers due to seroconversion (Figure 2). Of the 4,411 titers abstracted, 318 titers were from sera of unexposed individuals, 175 from the acute phase after exposure, 742 from convalescence, 2,326 from late convalescence and 850 from very late convalescence. Primary DENV exposures resulted in 2,248 (51%) titers, 832 (19%) titers were from secondary exposure, 80 (2%) from tertiary exposure, 318 (7%) from unexposed sera, and 933 (21%) from individuals with unknown infection histories. The study populations ranged from 1-year old children to 90-year old adults residing in at least 12 countries, half of which were in Asia (Table 1). Vaccination studies occurred mainly in the United States (n = 5) but also in Thailand (n = 2) and Australia (n = 1). Sun et al. (2009) reported titers from the largest number of individuals (n = 71) (Figure 2 and Table 3) [48].

### Variation in laboratory methods

Twenty-nine articles (90%) reported DENV1 PRNT titers, nine (28%) of which did not report the strains used (Table 2). The Hawaii, 16007, and Mochizuki strains were used in multiple articles and six additional strains were reported in one article each (indicated by “Other” in Tables 2 and 3). Of these 29 articles, three (10%) tested multiple DENV1 strains. Three articles did not test against DENV1 strains (indicated by “n/a”). Seventeen strains of DENV2 were tested in thirty articles and two articles (7%) reported PRNT titers against multiple strains. Titers against DENV2 strain 16681 were reported most frequently (n = 7 articles) followed by New Guinea C (n = 4), New Guinea B (n = 3), and PR-159 (n = 2). Two articles did not report PRNT titers against DENV2 strains. Titers against DENV3 strains were reported in 31 articles with strain H87 used in nine articles (28%). Seventeen different DENV3 strains were reported with two articles (6%) testing multiple DENV3 strains. Eight DENV4 strains were used in 27 studies and one article reported testing multiple strains. H241 was used most frequently (n = 9 articles), followed by 4328-S (n = 3) and Dominica/814669 and 1036 reported in 2 articles each.

The cell lines, neutralization percentages, and concentration of virus added to the PRNT were highly variable between articles (Table 1). Plaque-forming units (pfu) and focus-forming units (ffu) (quantities of viral particles differentiated by infection of neighboring cells in an assay) varied widely and were combined with varying volumes of sera. LLC-MK_2_ cells were used in fifteen articles (47%) while six and nine articles (19% and 28%) reported using Vero or BHK-21 cells, respectively. Two articles (6%) did not report the cell lines used and one article reported using both BHK-21 and LLC-MK_2_ cells for the purposes of comparison [31]. Twenty-three articles reported neutralization percentages of 50%, while four articles reported 70%, two articles reported 80%, and one article reported 90%. One article compared neutralization percentages of 50% and 70% [31] and three articles did not report the neutralization percentage used [27,30,43].

### Patterns of reported PRNT titers in primary and secondary exposure

**Figure 3:**
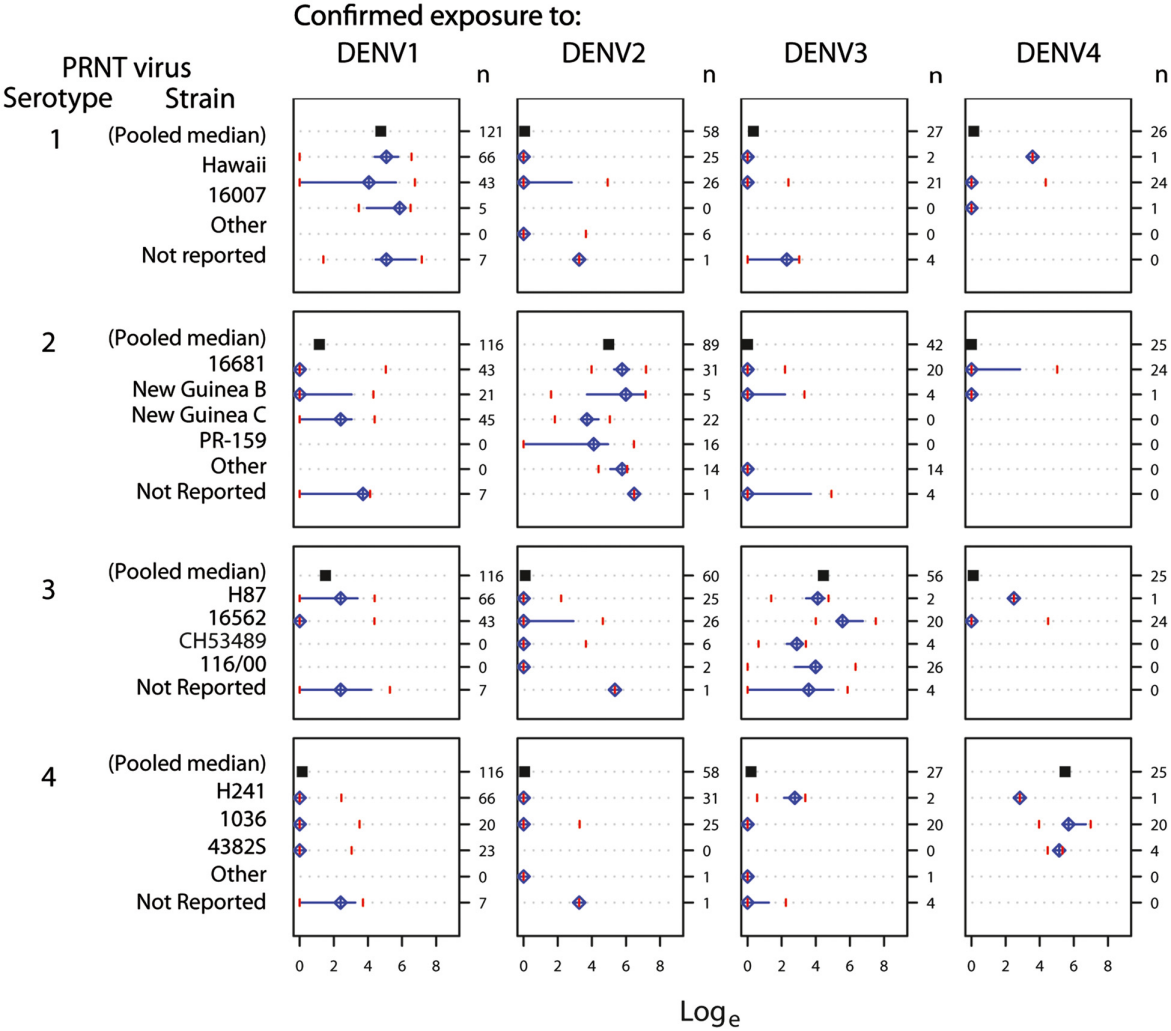
**Matrices of natural logarithm-transformedPRNT titers from primaryDENV exposures stratified bystrain.** The “n” on the upper right of each exposure column represents the number of titers, filled squares indicate the pooled median log titer for all strains within that particular box, diamonds with crosshairs represent median log titers for strains, horizontal lines span the 25th-75th percentiles (interquartile range), and vertical lines mark the 5th and 95th percentiles of the data.

Titers were excluded from the pooled analysis if the exposure serotype was not reported (n = 786), if exposure occurred via multitypic vaccine suspension (n = 944) or if titers were from unexposed sera (n = 74), leaving 1,615 primary and secondary exposure titers. Among 987 titers from primary exposures, titers to the exposure serotype were consistently high, and there was low reactivity to non-exposure serotypes (Figure 3). When sera from confirmed DENV1 exposures were tested against DENV1 strains, the pooled median log titer was 4.75 (standard error [SE]: 0.049) while testing DENV1-exposed sera with DENV2, DENV3, and DENV4 strains produced pooled median log titers ranging from 0.14-1.51. Homotypic testing with DENV2, DENV3, and DENV4 resulted in pooled median log titers of 4.98 (SE: 0.103), 4.45 (SE: 0.119) and 5.49 (SE: 0.118), respectively, while heterotypic testing produced titers ranging from 0–0.288.

**Figure 4:**
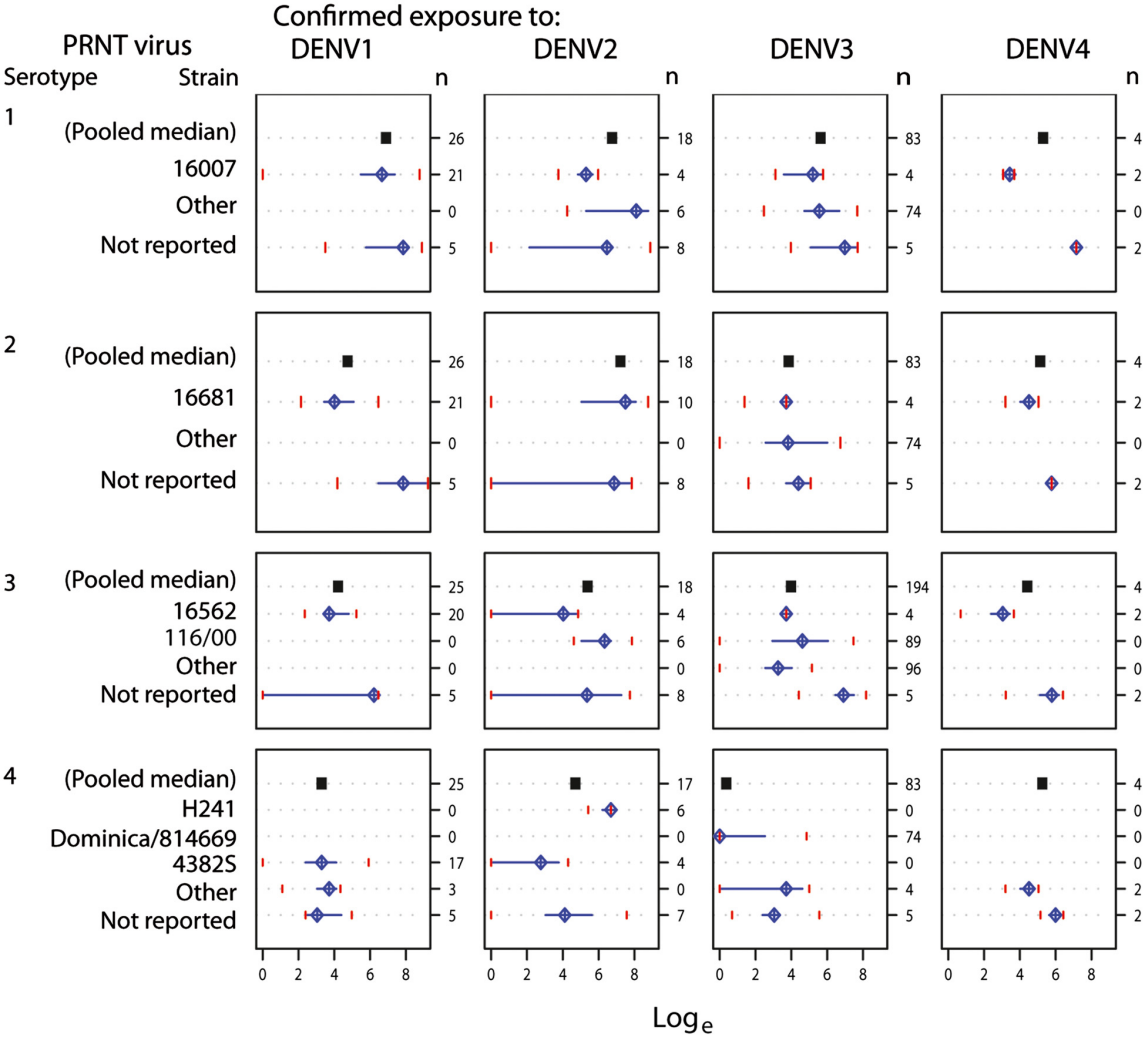
**Matrices of natural logarithm-transformedPRNT titers from secondaryinfections stratified by strain.** The “n” on the upper right of each exposure column represents the number of titers, filled squares indicate the pooled median log titer for all strains within that particular box, diamonds with crosshairs represent median log titers for strains, horizontal lines span the 25th-75th percentiles (interquartile range), and vertical lines mark the 5th and 95th percentiles of the data.

In contrast, secondary DENV exposures (n = 628 observations) produced high heterotypic titers, and testing with the exposure serotype did not always generate the highest PRNT titer (Figure 4). Pooled median log titers were highest when tested against the exposure serotype for DENV1 (6.89 [SE: 0.094]) and DENV2 (7.36 [SE: 0.042]) compared to non-exposure serotypes. For DENV3 exposure, however, the pooled median log titer from DENV3 testing (3.98 [SE: 0.059]) was lower than titers produced by DENV1 testing (5.63 [SE: 0.039]). Secondary DENV4 exposures showed a similar trend of high pooled median log titers against cross-serotypes, but few observations (n = 16) yielded uninformative statistical comparisons. As a result of these observations, the PRNT titer resulting from testing after secondary exposure was assumed to be independent of the secondary exposure serotype in the hierarchical models.

For the hierarchical models, titers were excluded if individuals were simultaneously exposed to more than one serotype via multivalent vaccination (n = 948) or the exposure serotype (n = 786), testing strain (n = 127) or exposure history (n = 911) were not reported. These criteria resulted in a total of 1,639 titers representing 929 individuals and 16 articles. The pattern of elevated titer against the exposure serotype was preserved in both models, with higher PRNT titers in sera tested against the exposure serotype compared to non-exposure serotypes after adjusting for other covariates (Table 4). For example, using estimates from model A, primary exposures to DENV2 tested against DENV2 result in an absolute titer of 9.17 (90% CrI: 4.84, 16.9) after adjusting for other covariates, whereas testing DENV2-exposed sera with DENV3 strains produces an absolute titer of 0.22 (90% CrI: 0.11, 0.44).

**Table 4:** **Point estimates and 90%credible intervals (CrI) fromthe log-linear hierarchical modelsof PRNT titers**

		**Model A**	**Model B**
**Intercept**		**Absolute Titer (90% CrI)**	
*Primary exposure*	*Testing serotype*		
Uninfected	DENV1	0.42 (0.16, 1.17)	0.43 (0.15, 1.29)
	DENV2	0.44 (0.15, 1.18)	0.43 (0.13, 1.35)
	DENV3	0.44 (0.16, 1.24)	0.44 (0.14, 1.26)
	DENV4	0.41 (0.14, 1.17)	0.42 (0.14, 1.19)
DENV1	DENV1	5.44 (2.91, 10.7)	15.1 (9.52, 23.8)
	DENV2	0.59 (0.31, 1.14)	1.19 (0.75, 1.89)
	DENV3	0.55 (0.28, 1.08)	1.17 (0.73, 1.85)
	DENV4	0.13 (0.06, 0.27)	0.29 (0.17, 0.48)
DENV2	DENV1	0.13 (0.06, 0.25)	0.46 (0.26, 0.81)
	DENV2	9.17 (4.84, 16.9)	26.1 (17.0, 40.9)
	DENV3	0.22 (0.11, 0.44)	0.65 (0.38, 1.10)
	DENV4	0.13 (0.06, 0.24)	0.37 (0.22, 0.63)
DENV3	DENV1	0.11 (0.05, 0.23)	0.36 (0.17, 0.77)
	DENV2	0.13 (0.06, 0.27)	0.32 (0.16, 0.62)
	DENV3	7.98 (3.96, 15.4)	32.6 (19.0, 55.2)
	DENV4	0.11 (0.05, 0.26)	0.30 (0.14, 0.65)
DENV4	DENV1	0.22 (0.11, 0.48)	0.52 (0.24, 1.07)
	DENV2	0.46 (0.21, 1.00)	0.98 (0.48, 1.99)
	DENV3	0.30 (0.13, 0.69)	0.72 (0.37, 1.43)
	DENV4	12.1 (5.93, 24.0)	29.8 (16.7, 52.4)
*Testing serotype among secondaryexposures*			
DENV1		26.5 (13.8, 53.8)	76.1 (43.0, 131)
DENV2		6.50 (3.26, 12.7)	17.6 (10.2, 30.3)
DENV3		5.46 (2.90, 10.4)	25.8 (15.3, 42.8)
DENV4		1.69 (0.79, 3.69)	2.63 (1.49, 4.57)
**Fixed Effect**		**Relative Titer (90% CrI)**	
10% increase in neutralization percentage		1.23 (1.14, 1.33)	
Vaccination		0.91 (0.70, 1.16)	0.89 (0.85, 0.94)
*Time category*			
Unexposed		Reference	Reference
1-11 days post-exposure (d.p.e.)		4.16 (2.57, 6.71)	2.86 (1.59, 5.01)
12-30 d.p.e.		8.51 (5.47, 13.2)	7.11 (4.46, 11.5)
31 -365 d.p.e.		5.57 (3.79, 8.24)	5.44 (3.68, 8.02)
>365 d.p.e.		3.13 (2.05, 4.81)	1.80 (0.98, 3.24)
**Random Effect**		**Relative Titer (90% CrI)**	
*Testing serotype*	*Testing strain*		
DENV1	16007	Reference	
	Hawaii	1.18 (0.79, 1.75)	
	Other DENV1 strains	0.97 (0.63, 1.50)	
DENV2	16681	Reference	
	New Guinea B	0.90 (0.53, 1.53)	
	New Guinea C	0.64 (0.40, 1.00)	
	PR-159	0.48 (0.25, 0.85)	
	Other DENV2 strains	0.90 (0.60, 1.31)	
DENV3	16562	Reference	
	H87	0.76 (0.49, 1.15)	
	116/00	1.33 (0.91, 1.96)	
	Other DENV3 strains	0.62 (0.43, 0.90)	
DENV4	1036	Reference	
	H241	0.73 (0.45, 1.17)	
	Dominica/814669	0.32 (0.16, 0.62)	
	4328-S	1.60 (1.02, 2.55)	
	Other DENV4 strains	1.13 (0.66, 1.88)	
**Variance Components**		**Proportion of the totalvariance (90% CrI)**	
Strain		8.04% (3.05%, 15.7%)	
Article			50.7% (30.8%, 71.6%)
**Deviance Information Criteria**		14910	14830

The two models account for variance in PRNT titers due to differences between strains (Model A) or articles (Model B). The average PRNT titer of sera collected from individuals with specific characteristics can be calculated as the product of the exponential of the coefficients listed. For example, using estimates from model A, the average titer for convalescent individuals with primary infections from DENV2, tested against DENV2 strain New Guinea B using a PRNT_60_ would be: 9.17 [primary DENV2 exposure, tested against DENV2] * 0.90 [tested against New Guinea B] * 1.23^6^ [PRNT_60_] * 8.51 [12–30 days post-exposure] = 243, or 1:243. Similarly, under the same conditions, secondary DENV2 exposure via vaccination would result in an average titer of 1:157 (6.50 [secondary DENV2 exposure, tested against DENV2] * 0.90 * 0.91 [vaccination] * 1.23^6^ * 8.51 = 157).

### Strain dependence of reported PRNT titers

Among individuals with confirmed primary DENV exposures, neutralization titers from each strain varied substantially within each serotype (Figure 3). For instance, individuals with primary DENV3 exposure had variability in unadjusted median log titers between strains ranging from 2.89 (IQR: 2.30, 3.23) for strain CH53489 to 5.56 (IQR: 5.21, 6.75) for strain 16562. Median log titers among individuals with secondary exposure also showed variability between strains; however, stratification by strain resulted in few observations and limited inference (Figure 4).

In the hierarchical models, strain variation accounted for 8.04% (90% CrI: 3.05%, 15.7%) of the variability in the PRNT titers after adjusting for other covariates (Table 4, Model A). While most strains produced relatively small effects on titers in comparison to the reference strains, others yielded more significant changes. Testing with DENV2 strain PR-159 and DENV4 strain Dominica/814669 yielded titers that were 0.48 [90% CrI: 0.25, 0.85] and 0.32 [90% CrI: 0.16, 0.62] that of titers from the respective reference strains, while DENV4 strain 4382-S produced 1.62-fold (90% CrI: 1.02, 2.55) increase in titer compared to the reference strain (Table 4, Model A). Additionally, accounting for strain in Model A reduced the effect of secondary exposure by 36-79% (Model A vs. B in Table 4).

### Other drivers of variation in PRNT titers

Time since exposure demonstrated a characteristic pattern of serologic response in both models, although we were unable to estimate the effect of acute DENV exposures due to a lack of titers fulfilling model inclusion criteria (Table 4). In Model A, convalescent DENV exposures (n = 339 titers) produced 8.51-fold (90% CrI: 5.47, 13.2) the mean titer of unexposed individuals (n = 177), decreasing to 5.57-fold (90% CrI: 3.79, 8.24) during late convalescence (n = 515) and 3.13-fold (90% CrI: 2.05, 4.81) during very late convalescence (n = 608). Each 10% change in neutralization percentage resulted in a 1.23-fold (90% CrI: 1.14, 1.33) change in titer (Table 4, Model A). Interestingly, titers resulting from vaccination (n = 433) were 0.91 (90% CrI: 0.70, 1.16) that of titers from natural exposure (Table 4). Cell line and virus concentration were evaluated as potential covariates but did not explain additional variation among titers as determined by increases in DIC values (data not shown).

Variation between articles (a proxy for inter-laboratory differences) produced 50.7% (90% CrI 30.8%, 71.6%) of the variation in PRNT titers not accounted for by other covariates (Table 4, Model B). Because of the association between article and laboratory methods, it is not possible to quantify how much of this variation is due to differences in strain and other aspects of the PRNT methods while also accounting for article variation. It is important to note that article-to-article variation may be inflated by differences in study population and other unmeasured covariates.

## Discussion

This systematic review of human PRNT titers to DENV demonstrated highly variable laboratory methods, particularly among viral strains. Despite analyzing 1,689 reported titers, the effects of using different strains were difficult to ascertain given the heterogeneity in laboratory methods. The original report on the development of a PRNT for DENV by Russell et al. (1967) was referenced frequently in the articles reviewed here [4], sometimes as the sole description of PRNT methods. However, most articles reported alterations to this protocol due to the identification of techniques or materials enhancing the assay’s performance in their laboratories. A large number of articles did not report information such as neutralization percentage, cell lines, the use of complement, and virus concentrations, making it impossible to fully adjust for these factors. In addition to the diverse array of materials and methods, heterogeneity in PRNT titers can also be induced by volume of virus added, serum dilutions prior to addition of virus, plaque counting techniques such as accounting for plaque overlap, and titer calculations [7], which were rarely described in the articles reviewed here.

Several additional factors may have influenced the PRNT titers collected here and differences observed between strains after adjusting for other factors in the models. First, the study populations described in these reports were from several different geographical areas (Table 3), which may affect not only strains to which an individual was exposed, but also strains against which their serum was tested. Secondly, there is likely an association between the testing strains and primary or secondary exposure based on each study’s inclusion criteria and laboratory methods. Lastly, the antibody response elicited by different infecting strains may vary in quality, duration and magnitude, highlighting antigenic differences between strains that call for further exploration. For example, Asian dengue strains have been noted to produce different serological reactions than American strains [49]. This may explain why DENV2 strain PR-159 and DENV4 strain Dominica/814669 produced relatively lower titers than Thai references strains.

Strain variation produced differences in median log titers within each serotype. After adjusting for several covariates, the choice of strain accounted for approximately 8% of the variation in titers, while variation between articles, which was used as a proxy for inter-laboratory variation, explained half of the variation in titers, suggesting comparability between laboratories is currently quite poor. The use of reference strains alone will not solve this issue, but the use of reference reagents or proficiency panels would lend validity to each laboratory’s results by offering a means of quality assurance and allow each laboratory to compare “in-house” materials and methods against a standard. Alternatively, algorithms that control for protocol variations would allow laboratories to continue with their preferred materials and methods but would adjust results for comparability.

Most often, PRNT titers are reported as the reciprocal of the serum dilution that shows a 50% reduction in the number of plaques produced by DENV exposure. While most studies use this neutralization level, higher neutralization percentages (e.g., PRNT_60_) have been used to increase specificity and prevent the detection of cross-neutralizing antibodies [18], but this must be weighed against decreased sensitivity [6]. Upon inclusion of neutralization percentage in the hierarchical model, an increase in titer was observed with each 10% increase in neutralization percentage. While we would expect a decrease in titer with higher neutralization percentage (e.g., 1:80 in PRNT_50_ versus 1:10 in PRNT_60_), this relationship may have been highly influenced by the few low titers reported in studies using higher neutralization percentages (e.g. PRNT_90_) and the many low titers reported in studies using PRNT_50_.

As titers were abstracted directly from the literature, our results are limited by published data. Titers are more likely to be reported and published if the findings are unusual or significantly different from previous reports. Also, relatively few titers (n = 177) were reported from unexposed individuals. Prior exposure to DENV was determined based on authors’ report, which may result in misclassification, and the primary exposures were unknown among individuals with secondary exposures. Original antigenic sin and variability in cross-reactive responses may have large effects on neutralization responses that are impossible for us to quantify without knowledge of primary exposures [34]. Furthermore, titers from individuals exposed to other flaviviruses were not included in this review. The exposure history of individuals to other flaviviruses was unknown in our sample and cross-reactions with non-dengue flaviviruses may be an additional factor to consider when analyzing PRNT data. The data were inadequate to estimate secondary exposure-test serotype pair effects in addition to effects for individual strains or articles. Lastly, the inclusion of reports published in English, Spanish and Portuguese reduced the potential for geographic selection bias, but may have excluded influential Asian studies.

## Conclusions

By synthesizing data from multiple sources, this analysis allows for between-laboratory and between-strain comparisons in addition to other factors that can influence PRNT titer variation. In-house optimizations of the PRNT that initially appear to produce minute changes may combine to create large differences when comparing results across multiple laboratories. These factors may have a significant impact on the neutralization capabilities of antibodies elicited in response to DENV exposure, hindering the ability to decipher immune protection and infection history. Despite well-known protocol variations, inadequate descriptions of materials and methods make inferential adjustments for these differences impractical. This requires improvement.

While we believe the PRNT provides a correlate of protection, the current methods do not take full advantage of quantitative results and render informal categorization of neutralization responses. Clinical endpoints will likely be used to assess vaccine efficacy but discrepancies in protection will require more thorough assessments of neutralization titers. Systematic characterization of antigenic similarities between strains will help clarify which strains are likely to induce immunogenicity and protection against other strains, aiding in vaccine strain selection. Nevertheless, as promising vaccine candidates arise, the lack of standardized assays among diagnostic and research laboratories will make unbiased inferences about vaccine-induced protection difficult. Sources of variation have important implications for vaccine testing and comparability. Prudent study design of a candidate vaccine will ensure testing in multiple geographic locations by highly similar materials and methods, but will immunogenicity of competing manufacturers’ vaccines determined by differing materials and methods be comparable? This has direct consequences for clinical decision making and policy guidelines. Establishing methods for inter-laboratory comparisons will help unravel the complex cross-reactions that characterize dengue virus exposures.

## Competing interests

The authors declare that they have no competing interests.

## Authors’ contributions

KRL and IRB conducted literature review. JL designed and performed statistical analysis. KRL and JL drafted the manuscript. DATC conceived of the review and contributed to the design and analysis. All authors read and reviewed the final manuscript.

## Pre-publication history

The pre-publication history for this paper can be accessed here:

http://www.biomedcentral.com/1471-2334/12/233/prepub

## Supplementary Material

Additional file 1 **Full hierarchical model specifications.** (DOC 565 kb) Click here for file
