# Supplementary material for: Variation in dengue virus plaque reduction neutralization testing: systematic review and pooled analysis
Source: BMC Infect Dis. 2012 Sep 28;12:233. doi: 10.1186/1471-2334-12-233 (PMC3519720; doi:10.1186/1471-2334-12-233)

**Additional File 1.**

We fit hierarchical models to the data to examine the effect of strain and article on the resulting titer estimates after adjusting for other factors that might affect PRNT titers. In primary infection, each PRNT titer
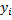
 was considered to depend on the infecting serotype,
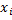
, and the testing serotype,
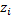
. In secondary infection,
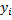
 was considered to depend only on the testing serotype,
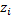
. In model A, random effects,
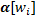
, with mean 0 and estimated variance was estimated to account for variation due to test strain (designated by
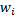
). Similarly, in model B, random effects
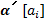
 were estimated to account for inter-article variation. In all models, values of
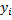
 less than the detectable threshold or greater than the maximum threshold in an article were censored (see Materials and Methods).

**Model A:**


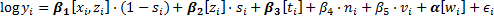


Where:


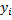
 is the measured PRNT titer for observation
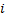


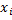
 is the confirmed infecting serotype for observation
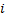


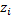
 is the test serotype for observation
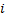


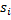
 is an indicator variable of whether observation
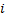
 is from an individual with secondary infection


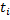
 is the categorical time since infection at which observation
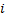
 was made


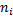
 is the neutralization percentage used as a stopping point in observation
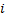


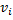
 is an indicator variable of if observation
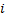
 represents an infection resulting from vaccination


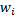
 is the strain used in observation
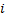


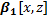
is the fixed effect of primary infection for individuals infected with serotype
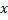
 being tested against serotype
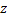
.


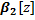
is the fixed effect of secondary infection when tested against serotype
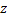


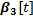
 is the fixed effect for individuals who are tested when the time since infection is category
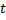


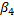
 is the fixed effect of neutralization percentage


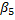
 is the fixed effect of vaccination


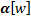
 is the random effect of strain used in the test, where
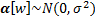


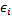
 is a normally distributed error term, such that
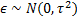


**Model B**


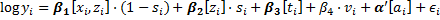


Where:


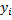
 is the measured PRNT titer for observation
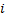


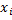
 is the confirmed infecting serotype for observation
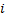


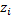
 is the test serotype for observation
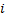


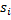
 is an indicator variable of whether observation
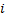
 is from an individual with secondary infection


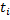
 is the categorical time since infection at which observation
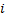
 was made


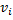
 is an indicator variable of if observation
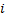
 represents an infection resulting from vaccination


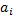
 is the article in which observation
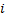
 was reported.


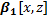
is the fixed effect of primary infection for individuals infected with serotype
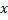
 being tested against serotype
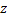
.


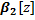
is the fixed effect of secondary infection when tested against serotype
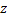


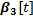
 is the fixed effect for individuals who are tested when the time since infection is category
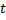


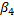
 is the fixed effect of vaccination


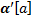
 is the random effect of article, where
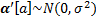


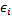
 is a normally distributed error term, such that
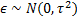

Supplement: Additional file 1 — Full hierarchical model specifications. (DOC 565 kb) [file 1471-2334-12-233-S1.doc]
